# Epigenetic Control of Macrophage Shape Transition towards an Atypical Elongated Phenotype by Histone Deacetylase Activity

**DOI:** 10.1371/journal.pone.0132984

**Published:** 2015-07-21

**Authors:** Mariana Cabanel, Camila Brand, Maria Cecilia Oliveira-Nunes, Mariela Pires Cabral-Piccin, Marcela Freitas Lopes, Jose Marques Brito, Felipe Leite de Oliveira, Marcia Cury El-Cheikh, Katia Carneiro

**Affiliations:** 1 Institute of Biomedical Science, Federal University of Rio de Janeiro, Rio de Janeiro, Brazil; 2 Carlos Chagas Filho Institute of Biophysics, Federal University of Rio de Janeiro, Rio de Janeiro, Brazil; St. Georges University of London, UNITED KINGDOM

## Abstract

Inflammatory chronic pathologies are complex processes characterized by an imbalance between the resolution of the inflammatory phase and the establishment of tissue repair. The main players in these inflammatory pathologies are bone marrow derived monocytes (BMDMs). However, how monocyte differentiation is modulated to give rise to specific macrophage subpopulations (M1 or M2) that may either maintain the chronic inflammatory process or lead to wound healing is still unclear. Considering that inhibitors of Histone Deacetylase (HDAC) have an anti-inflammatory activity, we asked whether this enzyme would play a role on monocyte differentiation into M1 or M2 phenotype and in the cell shape transition that follows. We then induced murine bone marrow progenitors into monocyte/macrophage differentiation pathway using media containing GM-CSF and the HDAC blocker, Trichostatin A (TSA). We found that the pharmacological inhibition of HDAC activity led to a shape transition from the typical macrophage pancake-like shape into an elongated morphology, which was correlated to a mixed M1/M2 profile of cytokine and chemokine secretion. Our results present, for the first time, that HDAC activity acts as a regulator of macrophage differentiation in the absence of lymphocyte stimuli. We propose that HDAC activity down regulates macrophage plasticity favoring the pro-inflammatory phenotype.

## Introduction

Inflammatory chronic pathologies such as arthritis and Crohn’s disease are complex processes characterized by an imbalance between the resolution of the inflammatory phase and the establishment of tissue repair. The main players in these inflammatory pathologies are bone marrow derived monocytes (BMDMs). BMDMs are continuously recruited to the inflammatory sites, where they proliferate and differentiate into macrophages, which then participate in the progression of wound healing [1].

During monocyte generation, the expression of the key transcription factor Pu.1, in progenitor hematopoietic cells, is associated to monocyte-commitment and responsiveness to GM-CSF (Granulocyte/Macrophage—Colony Stimulating Factor). This interaction triggers a sequence of events where the progenitor cell first differentiates into a monocyte, and then into an M1 inflammatory macrophage [2,3,4,5,6].

Histone Deacetylases (HDACs) are enzymes classically described as belonging to four different families, depending on their cellular location and HDAC inhibitor (HDACi) sensitivity [7]. HDACs are crucial chromatin remodelers that act by controlling the acetylation levels of nucleossomal histones, leading to transcriptional repression [8], which have been implicated in key physiological pathways [9], embryonic development [10] and regeneration [11]. It has been shown that HDAC activity is crucial for the pro-inflammatory phenotype, which occurs in synergy with the GM-CSF cell effect [12]. The blockage of HDAC activity has been extensively explored as anti-cancer therapy and also as anti-inflammatory drug in many clinical trials [12,13,14].

In addition to the molecular markers and cytokine secretion profile commonly used to identify macrophage sub-populations, pro-inflammatory M1 macrophages can also be distinguished from M2 macrophages by the classical pancake-like shape of the former [15]. Furthermore, pro-inflammatory M1 macrophages can have their function modulated by changing their shape. For instance, using micropatterning McWorther et al. have shown that macrophage elongation itself is tightly correlated to a shape transition from a pro-inflammatory M1 phenotype into an anti-inflammatory M2 phenotype. How monocyte differentiation is modulated to give rise to specific macrophage subpopulations (M1 or M2) that may either maintain the chronic inflammatory process or lead to wound healing is still unclear.

Considering that HDAC inhibitors have an anti-inflammatory activity, we asked whether this enzyme would play a role in monocyte differentiation into M1 or M2 phenotypes and in the cell shape transition that follows. In this study, we generated BMDMs cultures in the presence of Trichostatin A (TSA), a class I and II HDACi [16]. Here we present evidence, for the first time, that HDAC acts as a modulator of murine macrophage elongation, which is associated to a mixed M1/M2 phenotype. This outcome occurs even under GM-CSF induction and in the absence of lymphocyte stimuli. A better understanding on the mechanisms underlying this functional macrophage modulation is a relevant step towards the development of new treatments for old inflammatory diseases.

## Material and Methods

### Animals

Inbred C57BL/6 male mice of 8-week old were obtained from the colony bred at UFRJ, Brazil. All mice procedures were performed in strict accordance with institutional guidelines. The protocol was approved by the local Institutional Animal Care and Use Committee (IACUC) named Ethics Committee on Animal Use of the Health Sciences Center of the Federal University of Rio de Janeiro, under protocol number 118/14. The animals were euthanized under carbon dioxide chamber and all efforts were made to minimize suffering.

### Bone marrow cell cultures

Bone marrow cells were harvested by flushing the femoral cavity with α-MEM (Sigma-Aldrich M0644) containing 5% of fetal bovine serum (FBS) and 100 U/ml penicillin and 100 μg/ml streptomycin (culture medium). After adherence for one hour on plastic substrate, 10^6^ cells were plated in 6-well culture plates (Corning) and the cells were exposed to four different medium conditions and were subdivided in respective groups: Culture medium group (cells were cultivated only in culture medium), TSA group (cells were cultured in culture medium plus 10nM of TSA, Sigma T8552), GM-CSF group (cells were cultured in culture medium plus 10ng/ml of GM-CSF, Peprotech 315–03) and TSA+GM-CSF group (cells were cultured in culture medium plus 10ng/ml of GM-CSF and 10nM of TSA). The cells in the supernatant were harvest after 24 and 48 hours for further cells analysis. The quantification was performed using Trypan blue in the Newbauer chamber to monitor cell viability [17]. Morphological analysis was done by centrifuging cells on glass slides in a cytospin and stained with May-Grünwald-Giemsa by Pappenheim technique. To access the adherent cell population present in the cell culture system we have used circular glass cover slips (12-mm diameter) in the bottom of the culture plate to harvest cells in 3 and 6 days of culture. The cells were fixed for 1 hr in formaldehyde vapors, stained as described above and mounted for further morphological analysis.

### Flow cytometry analysis of myeloid progenitors cells

Aliquots of non-adherent cells from the experimental groups were harvested and incubated with the Fc blocker antibody, produced by clone 2.4G2 (obtained from the Rio de Janeiro Cell Bank, Inmetro, Rio de Janeiro Brazil), for 10 min before adding specific monoclonal antibodies anti-CD11b (Mac-1)-FITC, Gr-1-PECy5.5 and c-kit-APC (all from BD Bioscience, San Jose, CA, USA) for flow cytometry analysis. One hundred thousand events were acquired in a flow cytometer (FACScalibur, Bioscience) using Cell Quest software and analyzed using WIND 2.9 software.

### Immunocytochemistry staining of adherent cells on coverslips

The adherents cells on coverslips were fixed in 4% paraformaldehyde in PBS for 15 min at room temperature (RT), washed three times for 5 minutes with PBS, permeabilized in PBS/Triton X 0,2% for 5 minutes and blocked in PBS 5% BSA for 30 minutes.

The Moma-2 antibody was directly conjugated with Alexa-488-conjugated (AbDSerotec USA MCA519A488) [18]. Immunostaining was performed using primary anti-F4/80 (AbD Serotec), arginase 1 (Santa Cruz) or iNOS (Abcam) antibodies in PBS 1% BSA followed by incubation with secondary antibody conjugated to Alexa-488 fluorocrome (Molecular Probes) or 546 fluorochrome (Molecular Probes). Cells were incubated with primary antibodies in PBS containing 1% BSA overnight, at 4°C followed by four washes with PBS for 5 min and incubated with secondary antibodies for 1 h at RT in the dark. After PBS washes, cells were incubated with DAPI for 10 min at RT and the slides were mounted in DAKO paramount and the staining was visualized with Leica TCS SP5 AOBS. Images were handled and treated using Fiji program.

### Real time PCR

Supernatant cells derived from 24 and 48 hours cell culture were harvested and mRNA was extracted with TRIzol (Life Technologies), precipitated in ethanol and reversed transcribed using random hexamers. Quantitative real-time reverse transcription polymerase chain reaction (qRT-PCR) with SYBR green for PU1, GATA1, CEBPα, was performed following the manufacturer’s instructions (Applied Biosystems). A PCR from each sample before reverse transcription confirmed the absence of genomic DNA. RNA levels were standardized by parallel qRT-PCR using primers to the housekeeping gene, GAPDH (IDT). Primers for qRT-PCR were as follows:

PU1 forward, 5’–AGAAGCTGATGGCTTGGAGC- 3’;

PU1 reverse, 5’–GCGAATCTTTTTCTTGCTGCC- 3’;

GAPDH forward, 5’–ACCACAGTCCATGCCATCAC—3’;

GAPDH reverse, 5’–TCCACACCCTGTTGCTGTA– 3’;

GATA1 forward, 5’–CAGAACCGGCCTCTCATC– 3’;

GATA1 reverse, 5’- TAGTGCATTGGGTGCCTGC– 3’;

CEBP-α forward, 5’–AACTCGCTCCTTTTCCTACCGA—3’;

CEBP-α reverse, 5’–ACAGACTCAAATCCCCAACACC- 3’;

The quantification of mRNA levels was performed using ΔΔCt method.

### Western blot

Cell lysis was done in RIPA buffer solution in the presence of protease inhibitors. The protein samples were separated by electrophoresis on 10% acrylamide gel and blotted to PVDF membrane. The immunoblotting assay blocking step was performed with 5% non-fat dry milk powder in 0.1% PBS Tween and incubated overnight with primary antibody for arginase 1 (Santa Cruz), iNOS (Santa Cruz) antibodies or alpha-tubulin (Sigma). The secondary antibody used was HRP-peroxidase (Life Technologies). The reaction was developed using Luminol (Thermo Scientific) and bands were quantified by Photoshop.

### ELISA

Cytokines IL-1β, IL-10, IL-12p70 and TNF-α, as well as chemokines CCL1, CCL17 and CXCL-13 were evaluated in culture supernatants by a sandwich ELISA, using pairs of specific mAbs, one of which was labeled with biotin (all from R&D Systems, except for TNF-α reagents from eBioscience). Reactions were developed with alkaline phosphatase-streptavidin (Invitrogen Life Technologies, Carlsbad, CA, USA) and ρ-nitrophenylphosphate substrate (Sigma-Aldrich), according to manufacturer’s instruction.

### Nitric Oxide

Supernatants were mixed with an equal volume of Griess reagent to determine nitrite content, as described [19].

### Statistical Analysis

GraphPad Prism (v6.0, La Jolla, CA) was used for unpaired Student’s *t*-test or two-way ANOVA analysis where appropriate. If the ANOVA produced a significant result, *post hoc* pair-wise comparisons were tested for significance in which the *P* value was adjusted (*P*_adj_ < 0.05) by the Sidak method for multiple comparisons among the individual groups. Results are presented as mean±SD and statistical significance was defined as *P*<0.05.

## Results and Discussion

### Pharmacological knockdown of HDAC affects myeloid cell differentiation

**Fig 1:**
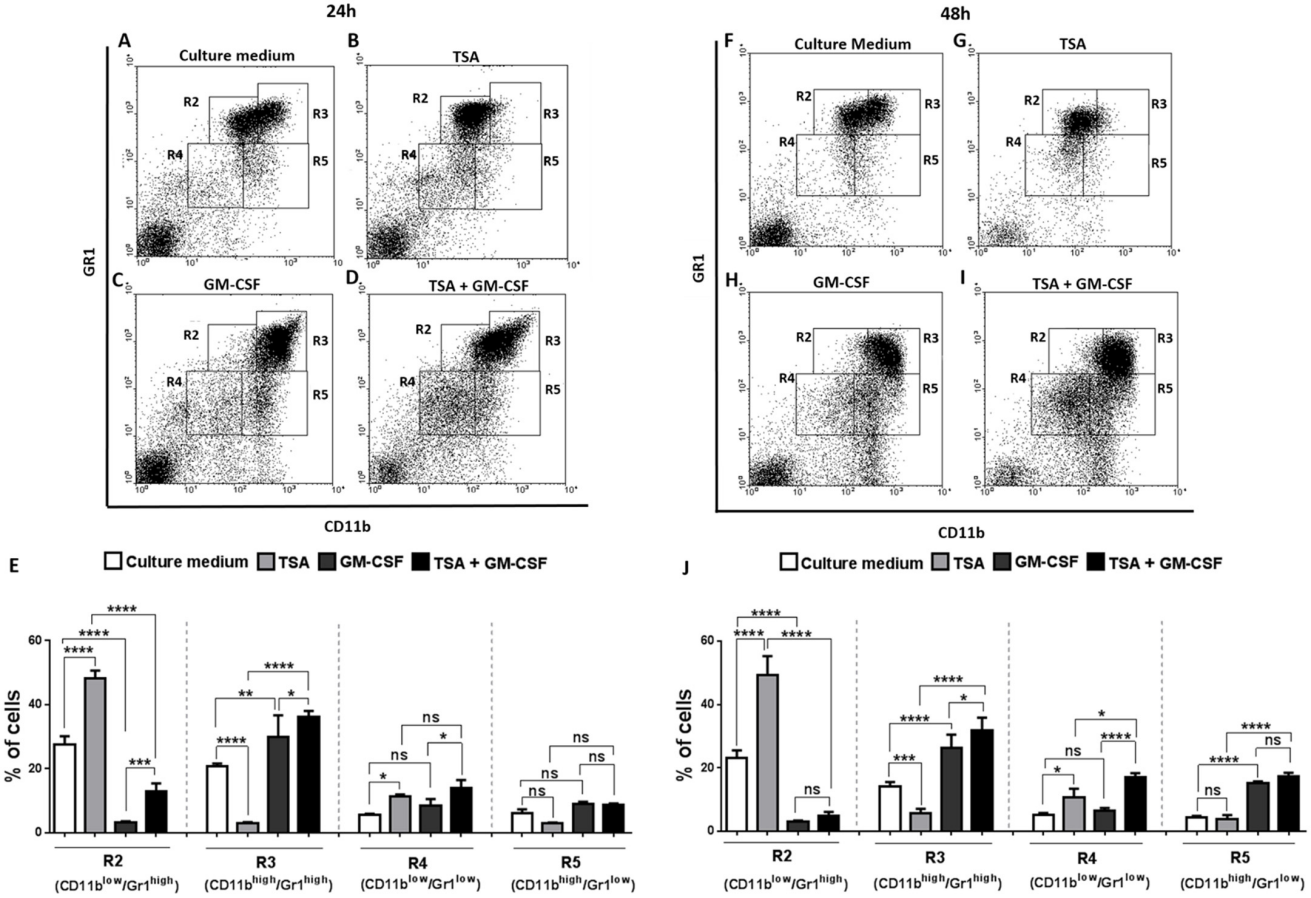
Inhibition of HDAC activity by TSA affects mice myeloid differentiation, favoring progenitors cells (R4) after 24 and 48 hours of culture. (A-D) After 24 h of culture, myeloid cells were analyzed with the phenotypic markers CD11b and Gr1. (E) Percentage of cells in the regions: R2 (Gr1^high^CD11b^low^), R3 (Gr1^high^CD11b^high^) R4 (Gr1^low^CD11b^low^) and R5 (Gr1^low^CD11b^high^) (n = 4 mice per group). (F-I) After 48 h of culture, myeloid cells were analyzed with the phenotypic markers CD11b and Gr1. (J) Percentage of cells in R2, R3, R4 and R5 regions. Data are means ± SD. Statistically significant differences, * p <0.05 by two-way ANOVA for repeated measurements followed by the Sidak test for correction of the p value.

Using flow cytometry, we observed, as expected, a decrease in immature cells CD11b^low^ Gr-1^high^ (R2) in 24 h, under treatment with GM-CSF, when compared to the untreated culture medium group (Fig 1A, 1C and 1E; S1 Fig). This result confirms that GM-CSF acts on the progenitor cells driving them into the myeloid differentiation pathway as we observed a shift from R2 to R3 (increased levels of CD11b). At the same time, a cell population characterized as CD11b^low^ Gr-1^low^ (R4) was observed in the TSA+ GM-CSF group (Fig 1D and 1E). This result was confirmed by the morphological aspects of GM-CSF treated cells that presented a segmented nucleus (Fig C arrowheads in S1 Fig). In contrast, TSA+GM-CSF group presented large progenitor cells, which may represent CD11b^low^ Gr-1^low^ phenotype (Fig D arrows in S1 Fig).

In 48 h, we observed an increase in the CD11b^high^ Gr-1^high^ (R3) population in the GM-CSF group, which are more mature cells than CD11b^low^ Gr-1^high^ (R2), when compared to the untreated culture medium group (Fig 1F, 1H and 1J; S1E and S1G Fig). In contrast, in the TSA+GM-CSF group, the cell population characterized as CD11b^low^ Gr-1^low^ (R4) increased, meaning that the predominance of progenitors, already seen at 24 h of culture, still remained at 48 h (Fig 1H, 1I and 1J; S1D and S1H Fig). We conclude that GM-CSF treatment induced macrophage-like cell development (Fig G arrowheads in S1 Fig) and TSA+GM-CSF treatment favored progenitor-like myeloid cells (Fig H arrowheads in S1 Fig).

**Fig 2:**
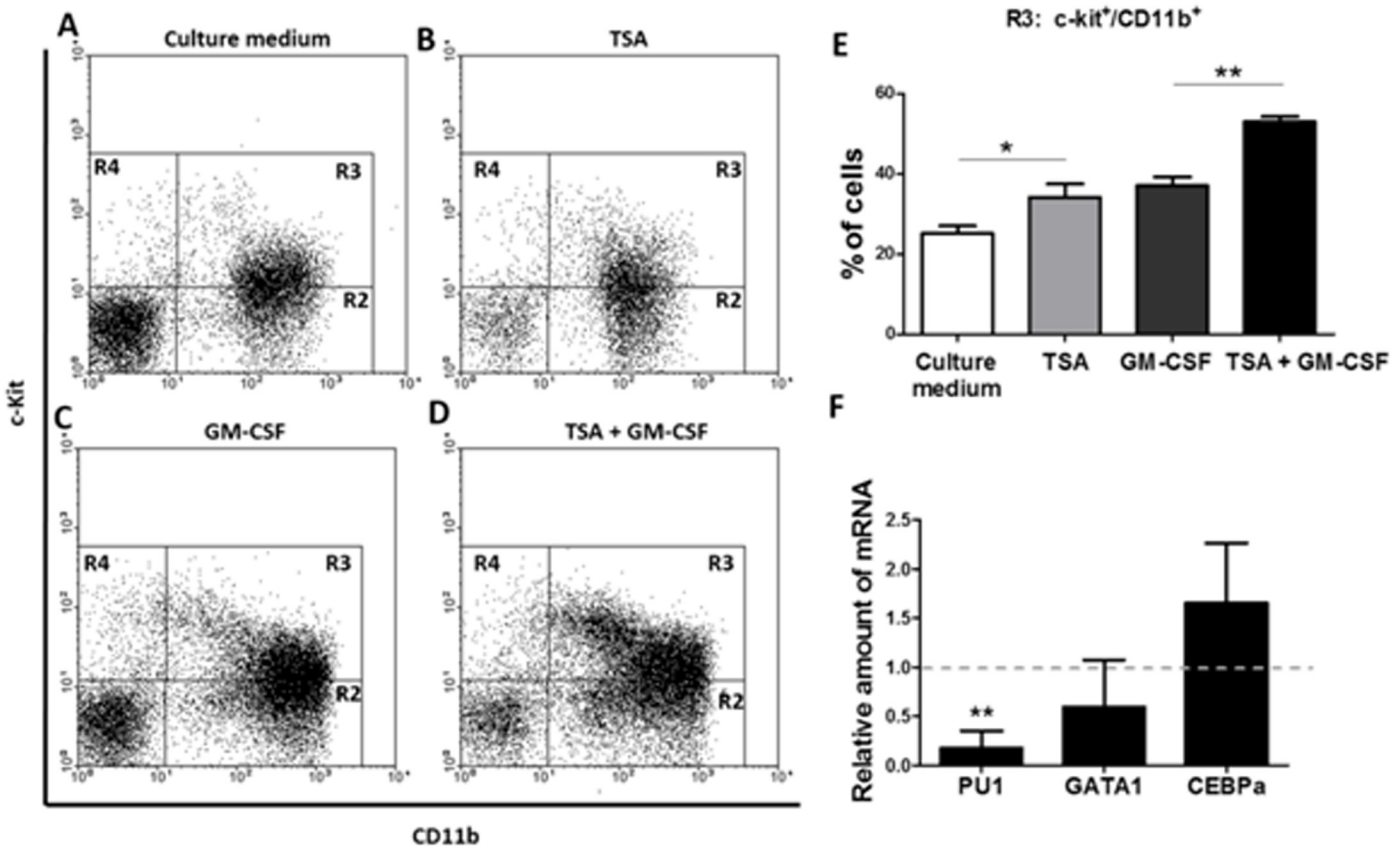
Inhibition of HDAC activity by TSA leads to increased c-kit positive myeloid progenitors cells and decreased PU.1 mRNA levels, after 48 hours of culture. (A-D) Analysis of the myeloid marker CD11b and the progenitor marker c-Kit. (E) Percentage of cells in the region R3 (c-Kit^+^ CD11b^+^) (n = 4 mice per group). (F) Relative amount of PU.1, GATA-1 and CEBP-α mRNAs, comparing between the TSA + GM-CSF and the GM-CSF cell cultures (1.0 represents the value for expression levels for GAPDH). Data are means ± SD. * p <0.05 by two-way ANOVA for repeated measures followed by the Sidak test for correction of the p value.

In agreement, we observed an increase in the percentage of c-Kit positive cells in the TSA+GM-CSF group, corroborating the role of TSA in the amplification of myeloid progenitors (Fig 2D and 2E). This increase in the percentage of c-Kit^+^ cells was followed by a significant decrease of PU.1 expression, if compared to expression levels observed in the GM-CSF group (Fig 2F). In addition, we also noticed the presence of clusters and colonies in the supernatant of the TSA+GM-CSF group observed in 5 days of culture, supporting the idea of an expansion of myeloid precursors (S2 Fig). Our findings indicate that the pharmacological knockdown of HDAC greatly interferes with myeloid differentiation, even though GM-CSF was added to the culture.

### Pharmacological knockdown of HDAC leads to a shape transition from the typical macrophage pancake shape into an elongated morphology

**Fig 3:**
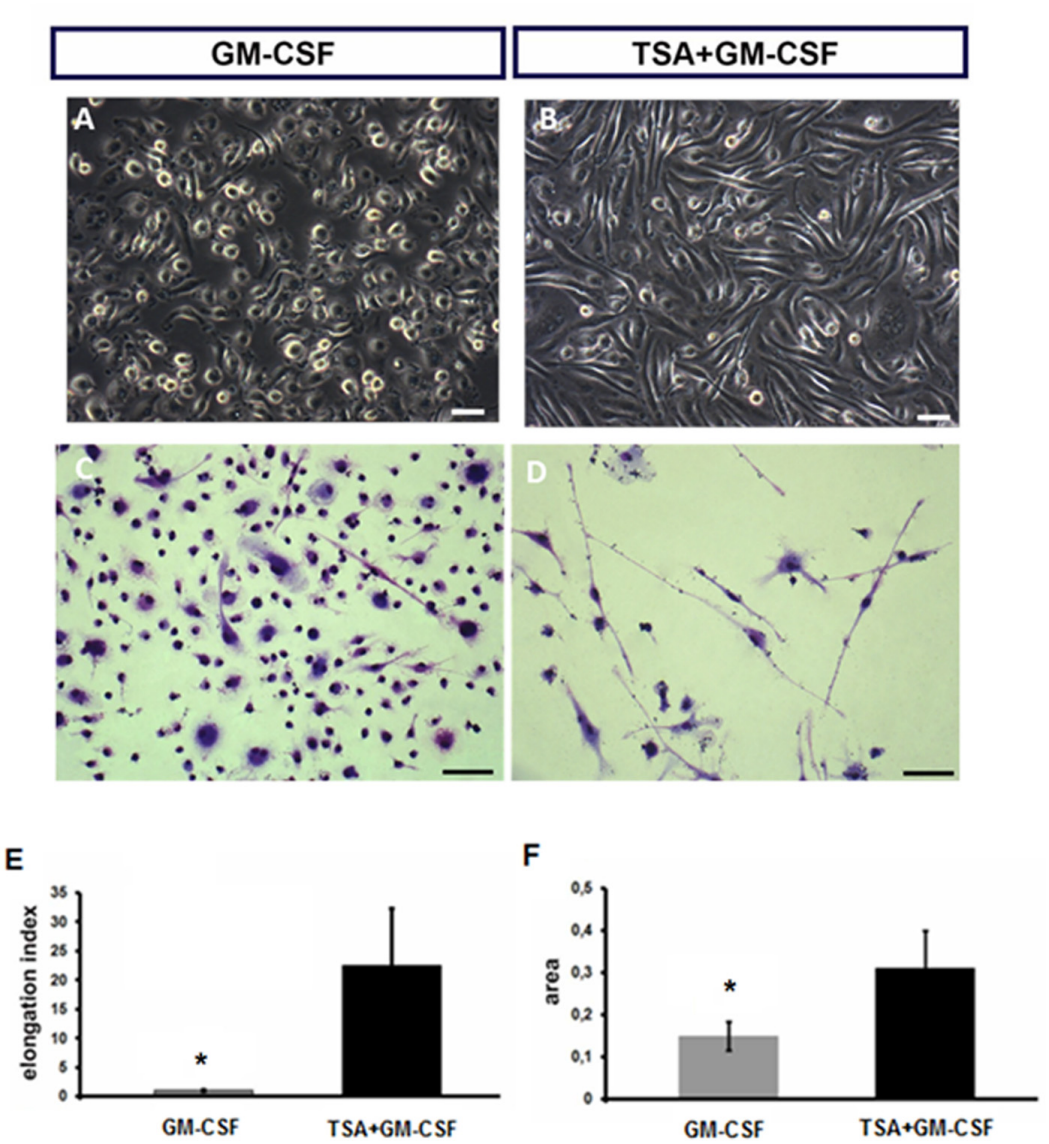
Inhibition of HDAC activity leads to a shape transition from the typical macrophage pancake shape into an elongated morphology after 11 days in culture. (A, B) Photomicrographs of bright field microscopy of cell culture. (C, D) Photomicrographs of the adherent cells stained by May-Grünwald and Giemsa, scale bars: 50μm. (A and C) Adherent cells in the GM-CSF group present a typical macrophage pancake shape. (B and D) Adherent cells generated in the TSA + GM-CSF group exhibit an atypical elongated morphology. Inhibition of HDAC activity increases the elongation factor (E) and the cell area (F) of adherent myeloid cells. (n = 3 per group). Data are means ± SD. * p <0.05 by Paired t test.

To evaluate the long-term pharmacological knockdown consequences of HDAC on monocyte lineage differentiation, we monitored the cultures for 11 days. We observed that the adherent cells in the TSA+GM-CSF group presented an atypical elongated morphology (Fig 3B and 3D), as demonstrated by the elongation index (Fig 3E) and increased cell area (Fig 3F). On the other hand, the monocytes stimulated by GM-CSF adhered on glass and showed a predominant and typical pancake-like shape of pro-inflammatory macrophages (Fig 3A and 3C). These results indicate that HDAC plays a role on the morphology of bone marrow monocyte lineage.

### The atypical elongated morphology of macrophages is related to a mixed M2/M1 phenotype

**Fig 4:**
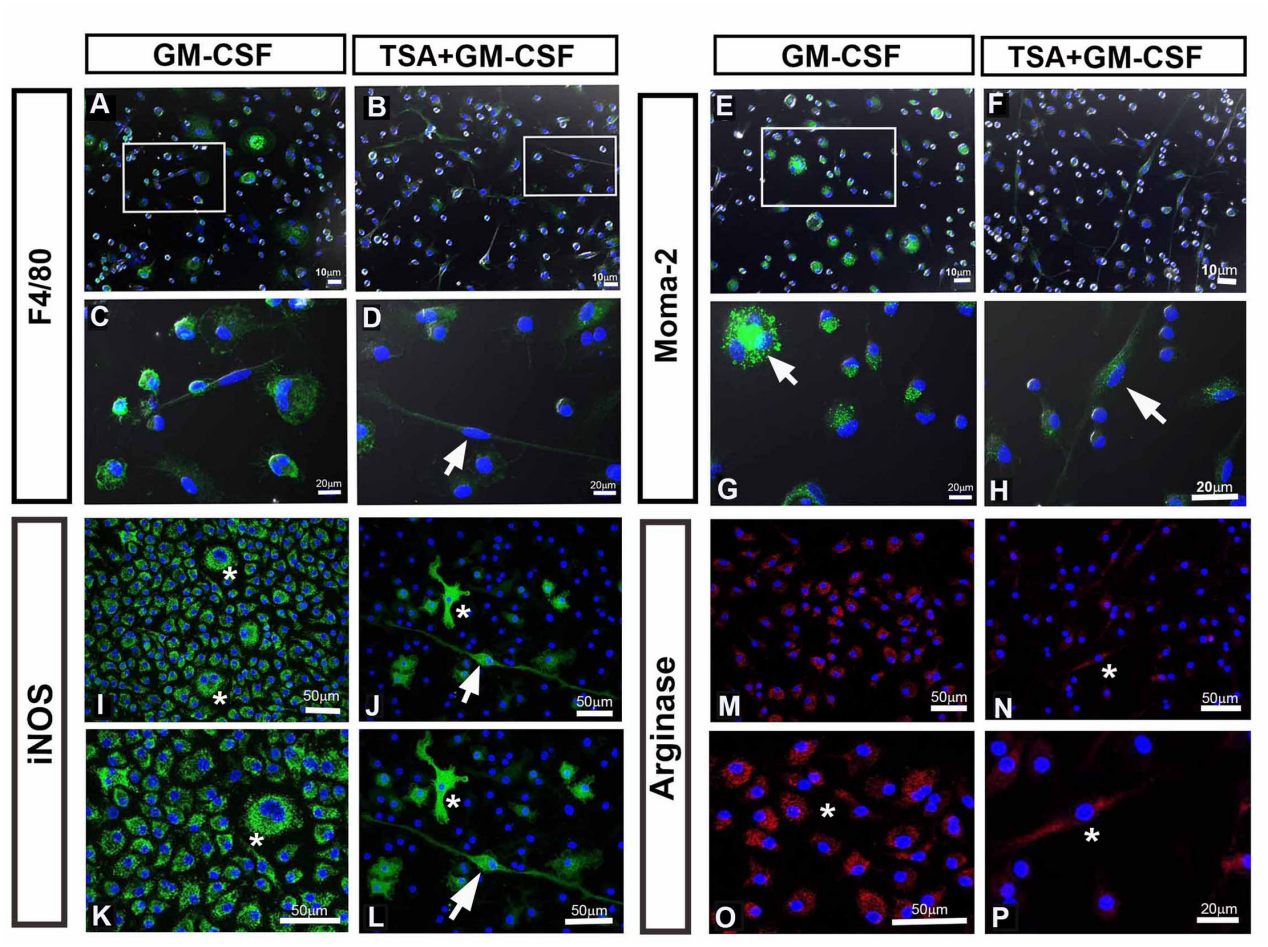
Inhibition of HDAC activity alters the phenotype of macrophages generated after 11 days of culture. **(A-H) Immunocitochemistry of activated-macrophage markers, F4/80 and Moma-2.** Pancake-like shape macrophages generated in the GM-CSF group are immunopositive for F4/80 (A and C) and Moma-2 (E and G). In the atypical elongated macrophages generated in the TSA + GM-CSF, the F4/80 and Moma-2 are down regulated (B, D, F and H—arrow). **(I-P) Immunocitochemistry of M1 macrophage marker iNOS, and M2 macrophage marker, arginase 1.** In the GM-CSF group, macrophages with pancake-like shape are immunopositive for iNOS (I and K) and for arginase 1 (M and O). The atypical elongated macrophage generated in the TSA + GM-CSF group are immunopositive for iNOS (J and L—arrow) and for arginase 1 (N and P—asterisk). Arginase 1 correlates to round cells in the GM-CSF group (M and O, asterisk) while in the TSA + GM-CSF group, the arginase 1 is associated only to elongated macrophages (N and P—asterisk).

Next, we investigated whether HDAC would work as a link between macrophage cell shape and an M1 or M2 phenotype profile. In the GM-CSF group we observed that the macrophages presenting the typical pancake-like shape were positive for both macrophage F4/80 and Moma-2, two pan-macrophage markers (Fig 4A, 4C, 4E and 4G; S3 Fig). However, macrophages presenting the atypical very elongated cell shape had both macrophage markers F4/80 and Moma-2 down regulated (Fig 4B, 4D, 4F and 4H; S3 Fig). This result indicates that HDAC pharmacological knockdown is related to a number of changes observed in these cells, from their shape to their expression pattern.

Considering that cells in the TSA+GM-CSF have a mixed morphology, including pancake-shape and atypical elongated cells, we asked whether this heterogeneous population present a bias towards M1 or M2 phenotype or a mixed M1/M2 phenotype. We found that pancake-shape GM-CSF cells were immunopositive for iNOS (Fig 4I and 4K) and arginase 1 (Fig 4M and 4O). While the former is a marker for M1 macrophage, the latter is a marker for M2. Interestingly, only elongated TSA+GM-CSF cells were immunopositive for iNOS (Fig 4J and 4L) and arginase 1 while the remaining pancake-shape macrophages did not stain for arginase 1 (Fig 4N and 4P). Protein quantification by western blot corroborates these findings showing that global levels of arginase 1 protein decreased in the TSA+GM-CSF group while iNOS levels remained stable (S4 Fig). These results, present evidence, for the first time, that HDAC acts as a regulator of macrophage differentiation as well as a modulator of macrophage elongation. This atypical elongated phenotype is correlated to a mixed M1/M2 phenotype. This outcome occurs even under GM-CSF induction indicating that the blockage of HDAC activity keeps the macrophage plasticity.

### HDAC activity blockage leads to a mixed M1/M2 profile of cytokine and chemokine secretion

**Fig 5:**
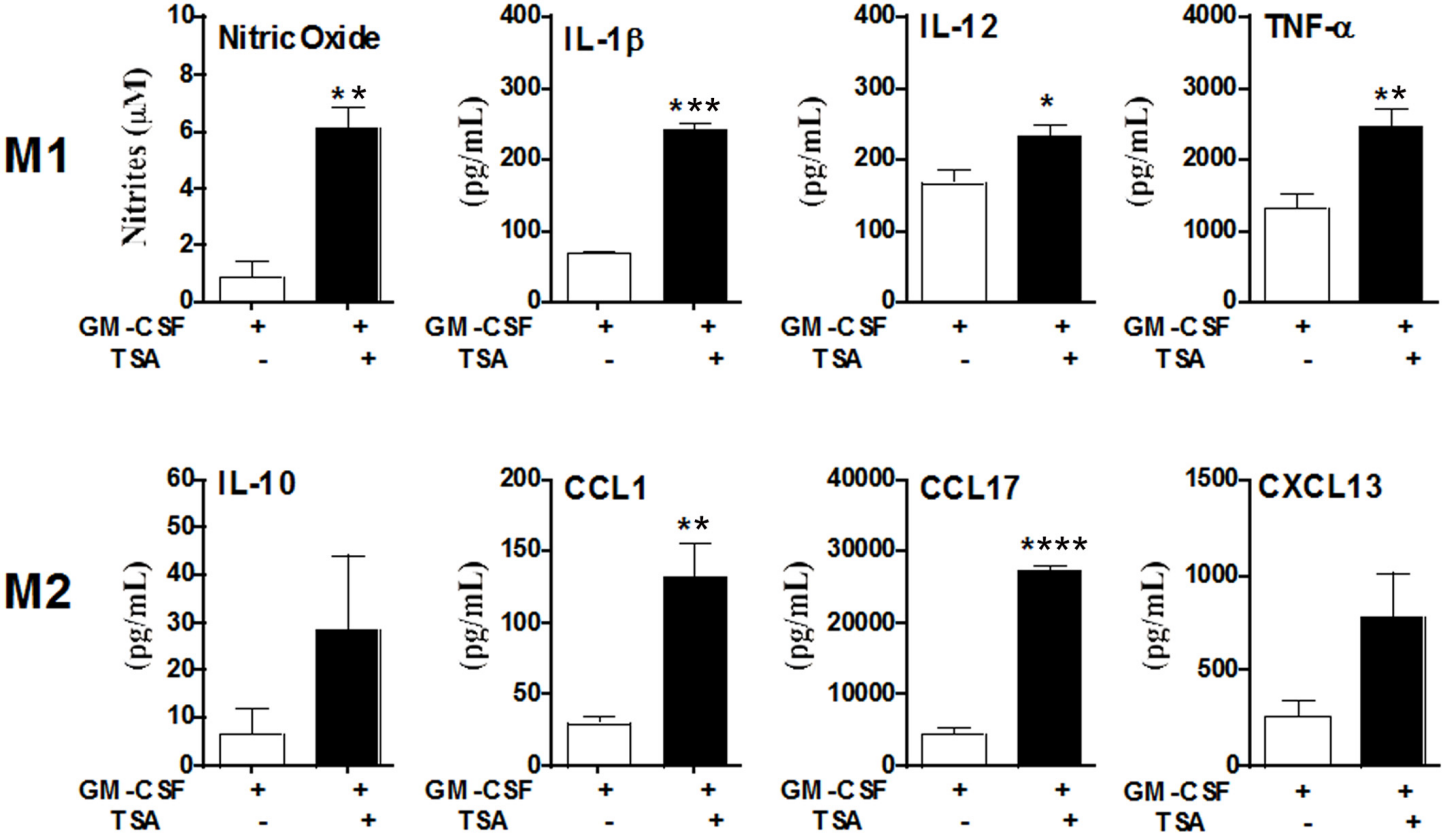
Inhibition of HDAC activity leads to a mixed M1/M2 secreation profile of cytokines and chemokines from macrophages. Secreted cytokines and NO production were accessed in supernatants from cultures treated with GM-CSF and TSA + GM-CSF groups. Nitric oxide (NO) production correlates with the concentration of nitrites produced in Griess reaction. Cytokine and chemokine concentrations were measured by sandwich ELISA assay. IL-1β, interleukin-1β; IL-12, interleukin-12; TNF-α, tumor necrosis factor-alpha; IL-10, interleukin-10; CCL1, chemokine (C-C motif) ligand 1; CCL17, chemokine ligand 17; CXCL13, chemokine (C-X-C motif) ligand 13. Data are means ± SEM. * p< 0,05 by unpaired t test, n = 6 for IL-12, TNF-α, CCL17 and CXCL13; n = 3 for NO, IL1-β, IL-10 and CCL1.

To better understand the role of HDAC activity on macrophage function we analyzed the cytokines and chemokines secreted by macrophages from GM-CSF and TSA+GM-CSF groups. ELISA assay showed a mixed profile of M1 and M2 cytokines and chemokines secretion in the GM-CSF group (Fig 5). In the TSA+GM-CSF group both M1 (NO, IL-1β, IL-12, TNF-α) and M2 functional markers (CCL1 and CCL17) were upregulated in the absence of further lymphocyte stimuli. Strikingly, the M2 marker CCL17, but not IL-10, was markedly increased upon treatment with TSA (Fig 5). Therefore, macrophage differentiation driven by GM-CSF can be modulated by HDAC activity blockage in order to keep phenotypic and functional plasticity represented by a mixed M2/M1 population by interfering with the terminal monocyte differentiation towards a pro-inflammatory phenotype.

Previous work showed that HDAC inhibition does not affect bone marrow macrophage terminal differentiation [20]. However, the populations of myeloid progenitors were not evaluated in the aforementioned study. In contrast, in our study we chose to use GM-CSF in order to expand a broader range of myeloid progenitors. Additionally, GM-CSF would drive M1 macrophage differentiation [21]. Indeed, there is an expansion of the myeloid precursors in cultures treated with TSA plus GM-CSF, (Figs 1 and 2), indicating that HDAC activity is required for maintaining the physiological status and timing necessary to allow macrophage progenitors to differentiate into the M1 phenotype. HDAC activity is also necessary to keep high levels of Pu.1 mRNA expression, than in turn, maintain the physiological levels of GM-CSF receptors on the cell surface, as previously described [22]. This supports the hypothesis that Pu.1, a key gene in macrophage differentiation, is an important HDAC target. Our results agree with previous studies showing a role for PU.1 in the expression of activated-macrophage markers, as well as their function in the inflammatory process [23].

These atypical elongated cells generated in the presence of TSA were positive for iNOs and arginase 1, while F4/80 and Moma-2, two pan-markers of macrophages, showed a weak staining if compared to that observed in the pancake-shape phenotype (Fig 4). This is in agreement with ELISA assay that showed a positive regulation of M1 (NO, IL-1β, IL-12, TNF-α) and M2 (CCL1 and CCL17) functional markers in the TSA+GM-CSF group, indicating lack of polarization. Therefore, our system supports the idea that the heterogeneity of M2/M1 is due, at least in part, to a mixed polarization of single cells. The results here presented strongly indicate the existence of an intrinsic plasticity of the myeloid progenitor uncovered by HDAC blockage.

Previous results published by [13,24] describe the requirement of HDAC3 for inflammatory gene expression program in macrophages, indicating that HDAC3 might be the main player in our system. However, it is important to keep in mind that other HDACs may have a role in our system. Indeed, Kittan et al have shown that depending on the cytokines added to cultures of blood monocytes, different HDACs are expressed (HDAC2, 5 and 9)[21].

McWhorter et al., 2013 have shown that macrophage cell shape is a critical landmark for M1/M2 phenotypes [15]. In this elegant study, the authors submitted macrophages to a mechanical force and demonstrated that elongation itself led to the expression of M2 markers, even when IL4 was absent. Our study is the first showing that macrophage shape transition is one of the outcomes observed when HDAC activity is blocked. Our study agrees with the findings of McWhorter et al., as we observed atypical elongated macrophages, even in the absence of TH2 cytokine stimuli. However, while McWhorter et al., 2013 achieved the elongated phenotype by applying an external mechanical force on already differentiated macrophages, we explored an intrinsic mechanism controlling macrophage differentiation that leads to an elongated atypical morphology which correlates to a mixed M1/M2 functional phenotype.

## Conclusions

We conclude that the pharmacological knockdown of HDAC greatly interferes with myeloid differentiation, even though GM-CSF was added to the culture. HDAC activity blockage led to the amplification of myeloid progenitors, that upon terminal differentiation driven by GM-CSF, displayed an elongated morphology and retained functional and phenotypical plasticity. In accordance, we propose that macrophages derived from bone marrow progenitors under HDAC activity blockage can develop mixed M1/M2 phenotypes.

Considering these findings, future studies are needed to evaluate the therapeutical potential of HDAC inhibition on the modulation of long lasting pathological conditions such as chronic inflammatory diseases (arthritis and Crohn’s disease) and in cancer therapies [25,26].

## Supporting Information

S1 Fig Cell morphology in the presence of GM-CSF and TSA after 24h and 48h of culture. Photomicrographs of cytospins stained with May-Grünwald and Giemsa after 24h and 48h of culture. Scale bars: 10μm. In the GM-CSF group, differentiated cells, neutrophils (C—arrow head) and macrophages (G—arrow head), predominate in culture. In the TSA + GM-CSF group, myeloid progenitors predominate in culture (D and H—arrows).
(TIF)

S2 Fig Inhibition of HDAC activity promotes expansion of myeloid progenitors, after 5 days in culture. (A-D) Photomicrographs of the cultures stimulated with GM-CSF in the absence of TSA (A and C) or its presence (B and D) after 5 days of culture. (B and D). The TSA + GM-CSF group exhibited an amplification of progenitors cells, as demonstrated by the presence of cell colonies in the culture supernatant. (A) and (C) Scale bars: 200μm. (B) and (D) Scale bars: 50μm. (n = 4 mice per group).
(TIF)

S3 Fig Inhibition of HDAC activity down regulates the two pan-macrophage markers, F4/80 and MOMA-2. Image J software was used to count cells grouped in three different brightness levels of fluorescence, (A) high, (B) medium and (C) low brightness of fluorescence. (D e E) The percentage of cells in each group in three independent images was quantified using Image J software.
(TIF)

S4 Fig Inhibition of HDAC activity decreases the level of arginase 1 protein and do not change the level of iNOS protein. (A) Representative Western Blot of iNOS, arginase-1, and α-tubulin obtained from GM-CSF and TSA+GM-CSF-treated myeloid cells. (B) Quantification of average across three separate experiments. Data are means ± SD. * p <0.05 by Paired t test, n = 3.
(TIF)

## References

[1] Mosser DM, Edwards JP (2008) Exploring the full spectrum of macrophage activation. Nat Rev Immunol 8: 958–969. 10.1038/nri2448 19029990 PMC2724991

[2] Shivdasani RA (1997) Stem cell transcription factors. Hematol Oncol Clin North Am 11: 1199–1206. 9443052 10.1016/s0889-8588(05)70489-5

[3] Shivdasani RA, Fielder P, Keller GA, Orkin SH, de Sauvage FJ (1997) Regulation of the serum concentration of thrombopoietin in thrombocytopenic NF-E2 knockout mice. Blood 90: 1821–1827. 9292514

[4] Shivdasani RA, Fujiwara Y, McDevitt MA, Orkin SH (1997) A lineage-selective knockout establishes the critical role of transcription factor GATA-1 in megakaryocyte growth and platelet development. EMBO J 16: 3965–3973. 9233806 10.1093/emboj/16.13.3965 PMC1170020

[5] DeKoter RP, Walsh JC, Singh H (1998) PU.1 regulates both cytokine-dependent proliferation and differentiation of granulocyte/macrophage progenitors. EMBO J 17: 4456–4468. 9687512 10.1093/emboj/17.15.4456 PMC1170777

[6] Kawamoto H, Ikawa T, Masuda K, Wada H, Katsura Y (2010) A map for lineage restriction of progenitors during hematopoiesis: the essence of the myeloid-based model. Immunol Rev 238: 23–36. 10.1111/j.1600-065X.2010.00959.x 20969582

[7] Gregoretti IV, Lee YM, Goodson HV (2004) Molecular evolution of the histone deacetylase family: functional implications of phylogenetic analysis. J Mol Biol 338: 17–31. 15050820 10.1016/j.jmb.2004.02.006

[8] Jenuwein T, Allis CD (2001) Translating the histone code. Science 293: 1074–1080. 11498575 10.1126/science.1063127

[9] Haberland M, Montgomery RL, Olson EN (2009) The many roles of histone deacetylases in development and physiology: implications for disease and therapy. Nat Rev Genet 10: 32–42. 10.1038/nrg2485 19065135 PMC3215088

[10] Carneiro K, Donnet C, Rejtar T, Karger BL, Barisone GA, Díaz E, et al (2011) Histone Deacetylase activity is necessary for left-right patterning during vertebrate development. BMC Dev Biol 11: 29 10.1186/1471-213X-11-29 21599922 PMC3113753

[11] Tseng AS, Carneiro K, Lemire JM, Levin M (2011) HDAC Activity Is Required during Xenopus Tail Regeneration. PLoS One 6: e26382 10.1371/journal.pone.0026382 22022609 PMC3194833

[12] Han SB, Lee JK (2009) Anti-inflammatory effect of Trichostatin-A on murine bone marrow-derived macrophages. Arch Pharm Res 32: 613–624. 10.1007/s12272-009-1418-4 19407980

[13] Chen X, Barozzi I, Termanini A, Prosperini E, Recchiuti A, Dalli J, et al (2012) Requirement for the histone deacetylase Hdac3 for the inflammatory gene expression program in macrophages. Proc Natl Acad Sci U S A 109: E2865–2874. 10.1073/pnas.1121131109 22802645 PMC3479529

[14] Drummond DC, Noble CO, Kirpotin DB, Guo Z, Scott GK, Benz CC (2005) Clinical development of histone deacetylase inhibitors as anticancer agents. Annu Rev Pharmacol Toxicol 45: 495–528. 15822187 10.1146/annurev.pharmtox.45.120403.095825

[15] McWhorter FY, Wang T, Nguyen P, Chung T, Liu WF (2013) Modulation of macrophage phenotype by cell shape. Proc Natl Acad Sci U S A 110: 17253–17258. 10.1073/pnas.1308887110 24101477 PMC3808615

[16] Yoshida M, Kijima M, Akita M, Beppu T (1990) Potent and specific inhibition of mammalian histone deacetylase both in vivo and in vitro by trichostatin A. J Biol Chem 265: 17174–17179. 2211619

[17] el-Cheikh MC, Borojevic R (1990) Extramedullar proliferation of eosinophil granulocytes in chronic schistosomiasis mansoni is mediated by a factor secreted by inflammatory macrophages. Infect Immun 58: 816–821. 2106496 10.1128/iai.58.3.816-821.1990 PMC258538

[18] Kraal G, Rep M, Janse M (1987) Macrophages in T and B cell compartments and other tissue macrophages recognized by monoclonal antibody MOMA-2. An immunohistochemical study. Scand J Immunol 26: 653–661. 3321409 10.1111/j.1365-3083.1987.tb02301.x

[19] Green LC, Wagner DA, Glogowski J, Skipper PL, Wishnok JS, Tannenbaun SR (1982) Analysis of nitrate, nitrite, and [15N]nitrate in biological fluids. Anal Biochem 126: 131–138. 7181105 10.1016/0003-2697(82)90118-x

[20] Rahman MM, Kukita A, Kukita T, Shobuike T, Nakamura T, Kohashi O (2003) Two histone deacetylase inhibitors, trichostatin A and sodium butyrate, suppress differentiation into osteoclasts but not into macrophages. Blood 101: 3451–3459. 12511413 10.1182/blood-2002-08-2622

[21] Kittan NA, Allen RM, Dhaliwal A, Cavassani KA, Schaller M, Gallagher KA, et al (2013) Cytokine induced phenotypic and epigenetic signatures are key to establishing specific macrophage phenotypes. PLoS One 8: e78045 10.1371/journal.pone.0078045 24205083 PMC3804553

[22] Laribee RN, Klemsz MJ (2001) Loss of PU.1 expression following inhibition of histone deacetylases. J Immunol 167: 5160–5166. 11673528 10.4049/jimmunol.167.9.5160

[23] Karpurapu M, Wang X, Deng J, Park H, Xiao L, Sadikot RT, et al (2011) Functional PU.1 in macrophages has a pivotal role in NF-kappaB activation and neutrophilic lung inflammation during endotoxemia. Blood 118: 5255–5266. 10.1182/blood-2011-03-341123 21937699 PMC3217408

[24] Mullican SE, Gaddis CA, Alenghat T, Nair MG, Giacomin PR, Everett LJ, et al (2011) Histone deacetylase 3 is an epigenomic brake in macrophage alternative activation. Genes Dev 25: 2480–2488. 10.1101/gad.175950.111 22156208 PMC3243058

[25] Tabas I (2010) Macrophage death and defective inflammation resolution in atherosclerosis. Nat Rev Immunol 10: 36–46. 10.1038/nri2675 19960040 PMC2854623

[26] Odegaard JI, Chawla A (2011) Alternative macrophage activation and metabolism. Annu Rev Pathol 6: 275–297. 10.1146/annurev-pathol-011110-130138 21034223 PMC3381938

