# Supplementary figures and images for: Epigenetic Control of Macrophage Shape Transition towards an Atypical Elongated Phenotype by Histone Deacetylase Activity
Source: PLoS One. 2015 Jul 21;10(7):e0132984. doi: 10.1371/journal.pone.0132984 (PMC4509762; doi:10.1371/journal.pone.0132984)

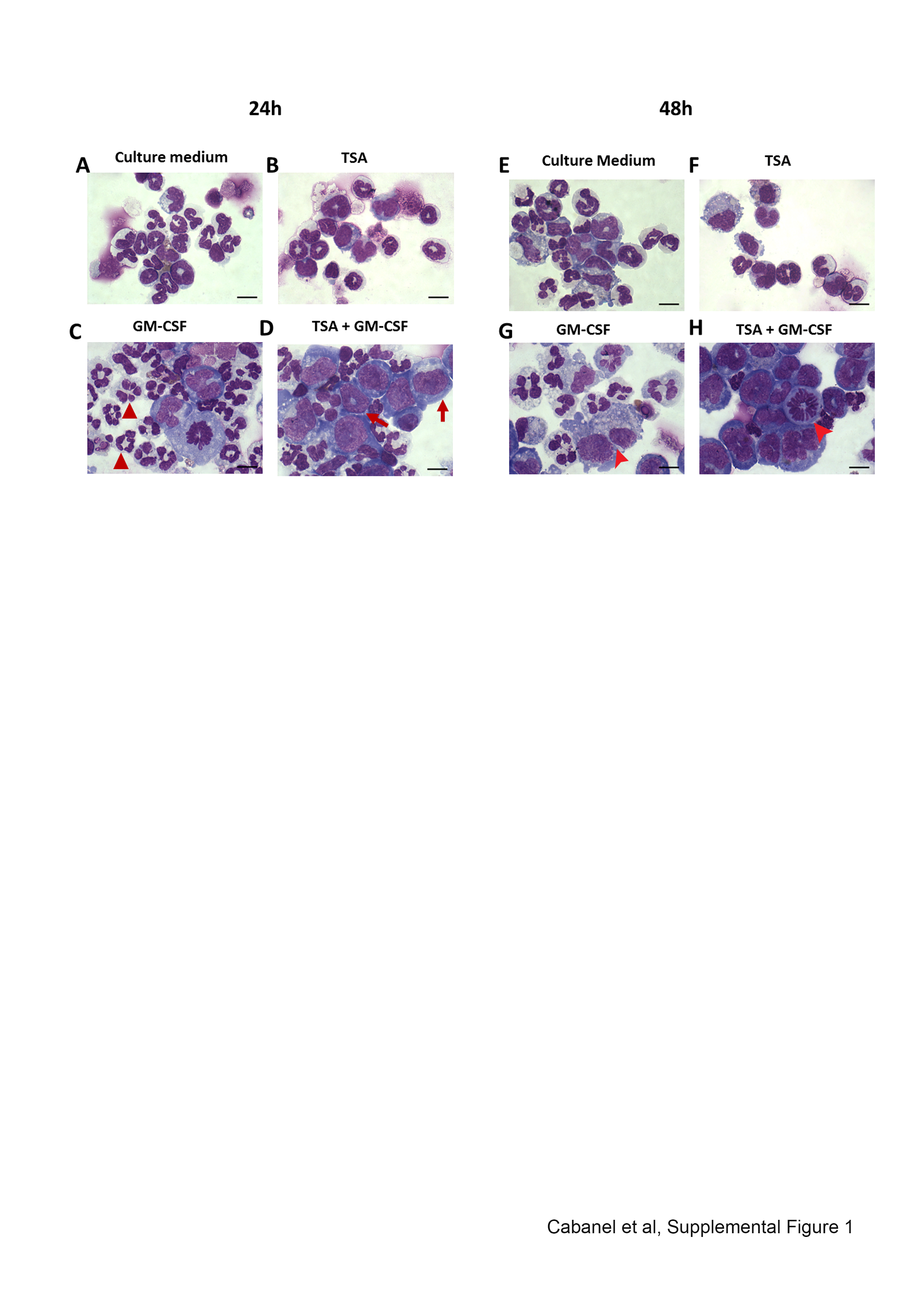

Supplement: S1 Fig — Photomicrographs of cytospins stained with May-Grünwald and Giemsa after 24h and 48h of culture. Scale bars: 10μm. In the GM-CSF group, differentiated cells, neutrophils (C—arrow head) and macrophages (G—arrow head), predominate in culture. In the TSA + GM-CSF group, myeloid progenitors predominate in culture (D and H—arrows). (TIF) [file pone.0132984.s001.tif]

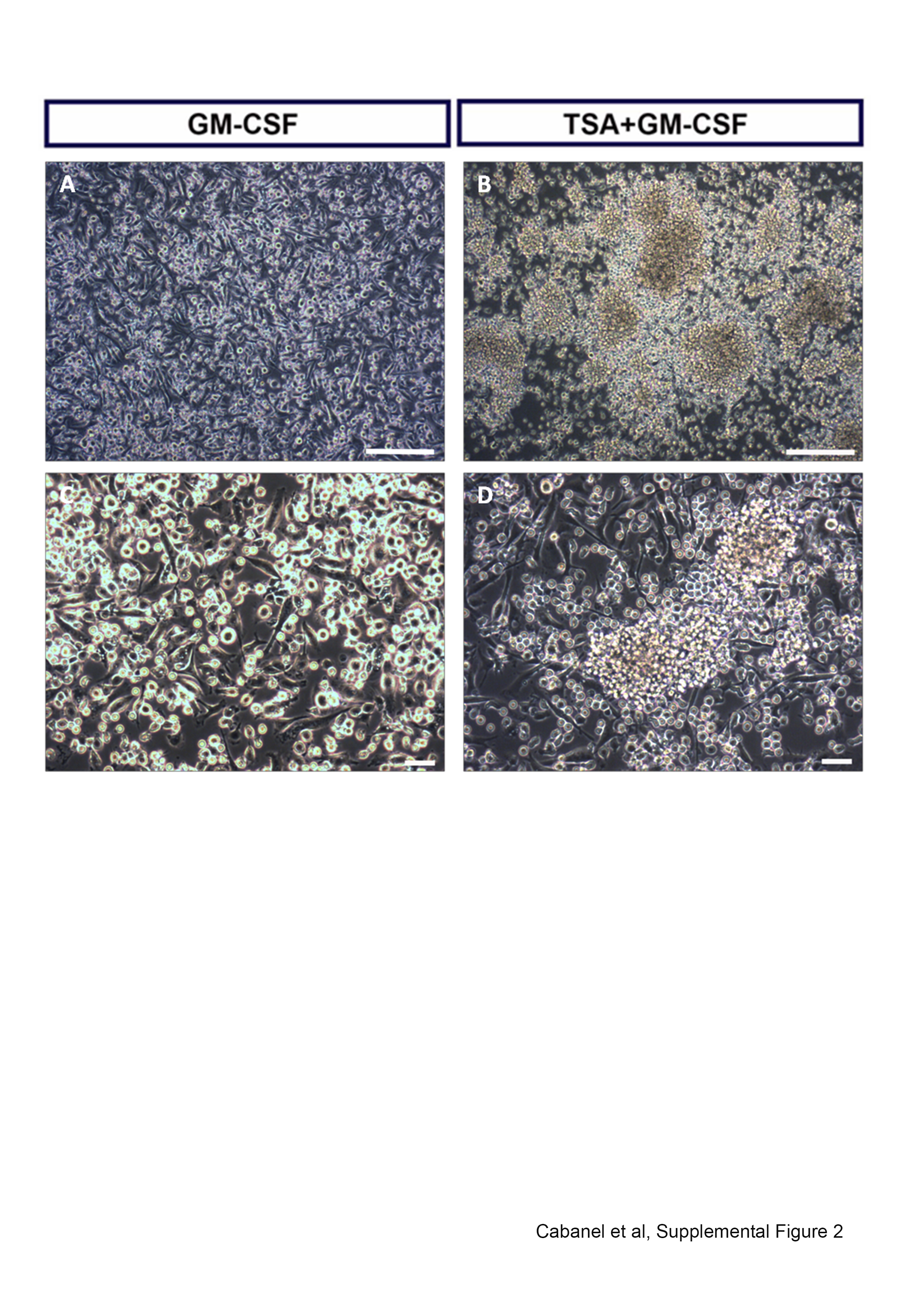

Supplement: S2 Fig — (A-D) Photomicrographs of the cultures stimulated with GM-CSF in the absence of TSA (A and C) or its presence (B and D) after 5 days of culture. (B and D). The TSA + GM-CSF group exhibited an amplification of progenitors cells, as demonstrated by the presence of cell colonies in the culture supernatant. (A) and (C) Scale bars: 200μm. (B) and (D) Scale bars: 50μm. (n = 4 mice per group). (TIF) [file pone.0132984.s002.tif]

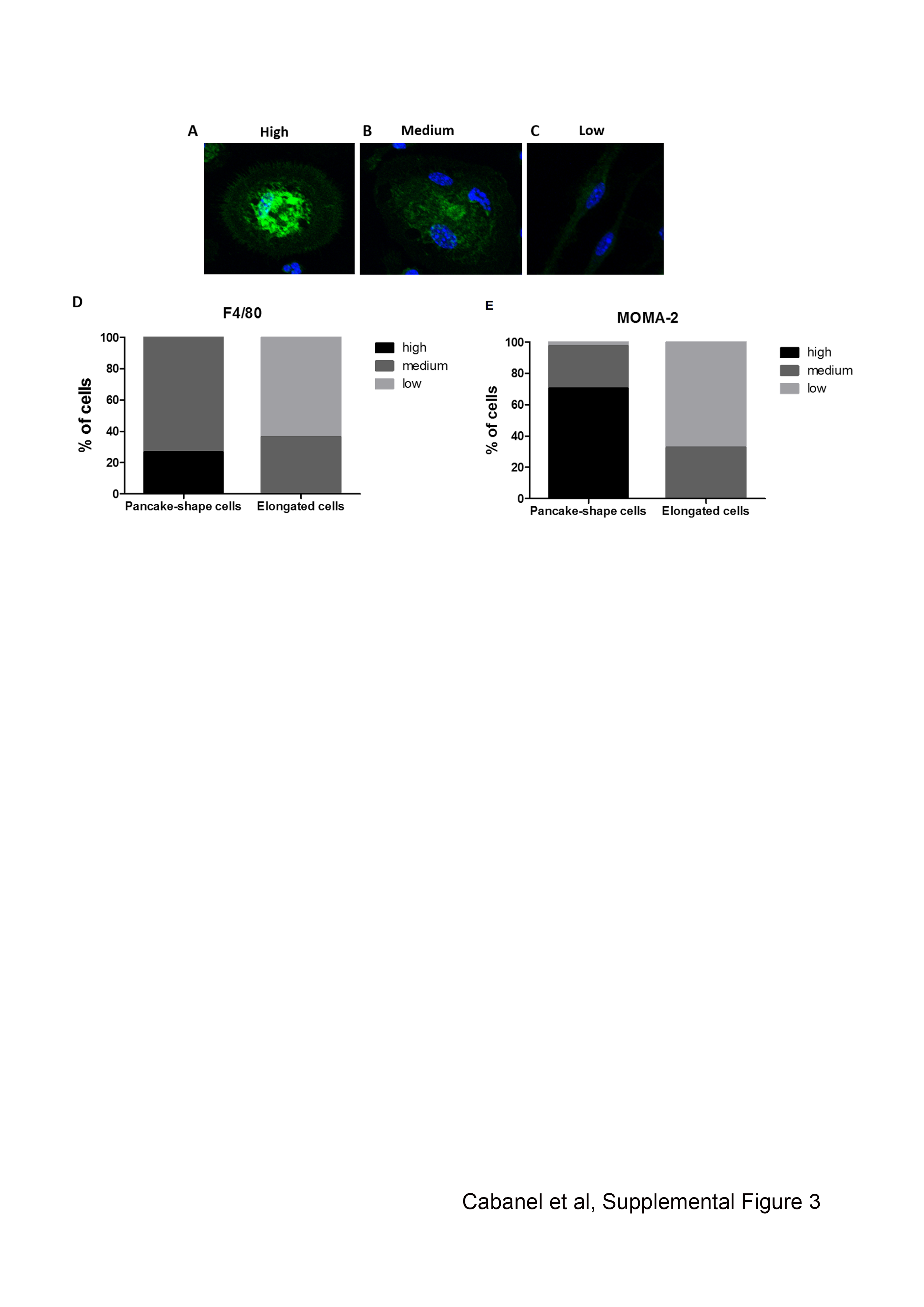

Supplement: S3 Fig — Image J software was used to count cells grouped in three different brightness levels of fluorescence, (A) high, (B) medium and (C) low brightness of fluorescence. (D e E) The percentage of cells in each group in three independent images was quantified using Image J software. (TIF) [file pone.0132984.s003.tif]

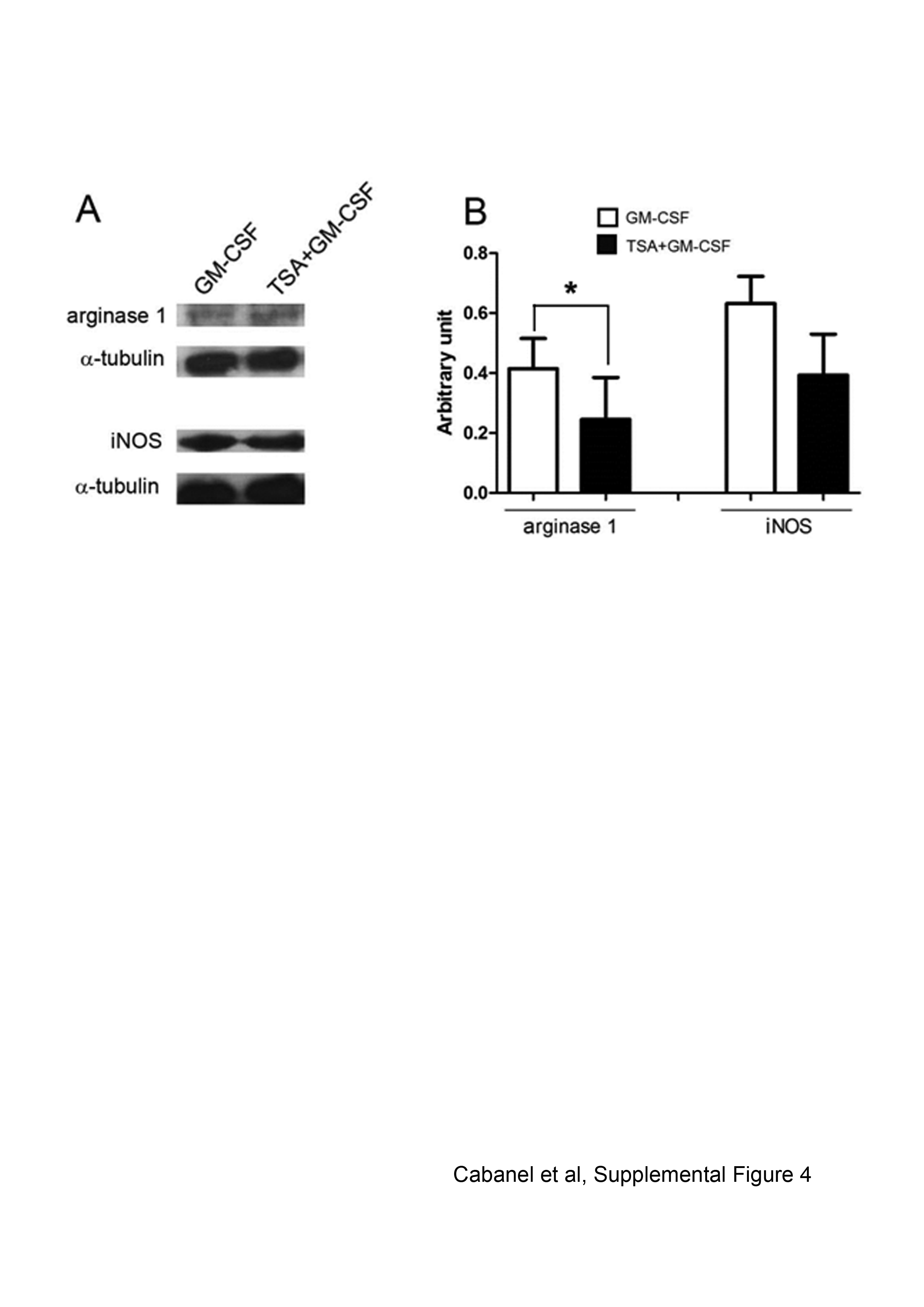

Supplement: S4 Fig — (A) Representative Western Blot of iNOS, arginase-1, and α-tubulin obtained from GM-CSF and TSA+GM-CSF-treated myeloid cells. (B) Quantification of average across three separate experiments. Data are means ± SD. * p <0.05 by Paired t test, n = 3. (TIF) [file pone.0132984.s004.tif]
